# Pharmacovigilance for rare diseases: a bibliometrics and knowledge-map analysis based on web of science

**DOI:** 10.1186/s13023-023-02915-y

**Published:** 2023-09-26

**Authors:** Mengdan Xu, Guozhi Li, Jiazhao Li, Huiyu Xiong, Suzhen He

**Affiliations:** 1 School of Clinical Pharmacy, Guangdong Pharmaceutical University, Guangzhou, Guangdong China; 2 NMPA Key Laboratory for Technology Research and Evaluation of Pharmacovigilance, Guangzhou, Guangdong China; 3 Center for ADR Monitoring of Guangdong, Guangzhou, Guangdong China; 4 The First Affiliated Hospital of Guangzhou Medical University, Guangzhou, Guangdong China

**Keywords:** Rare diseases, Pharmacovigilance, Orphan drugs, Knowledge-map, CiteSpace, Research hotspots

## Abstract

**Objectives:**

The aims of this paper is to search and explore publications in the field of pharmacovigilance for rare diseases and to visualize general information, research hotspots, frontiers and future trends in the field using the bibliometric tool CiteSpace to provide evidence-based evidence for scholars.

**Methods:**

We searched the Web of Science Core Collection (WoSCC) for studies related to pharmacovigilance for rare diseases, spanning January 1, 1997-October 25, 2022. CiteSpace software was utilized to discuss countries/regions, institutions, authors, journals, and keywords.

**Results:**

After screening, a total of 599 valid publications were included in this study, with a significant upward trend in the number of publications. These studies were from 68 countries/regions with the United States and the United Kingdom making the largest contributions to the field. 4,806 research scholars from 493 institutions conducted studies on pharmacovigilance for rare diseases. Harvard University and University of California were the top two productive institutions in the research field. He Dian of the Affiliated Hospital of Guizhou Medical University and Peter G.M. Mol of the University of Groningen, The Netherlands, were the two most prolific researchers. The Cochrane Database of Systematic Reviews and the New England Journal of Medicine were the journals with the highest number of articles and co-citation frequency respectively. Clinical trial, therapy and adverse event were the top three most cited keywords.

**Conclusions:**

Based on keywords co-occurrence analysis, four research topics were identified: orphan drug clinical trials, postmarketing ADR surveillance for orphan drugs, rare diseases and orphan drug management, and diagnosis and treatment of rare diseases. Immune-related adverse reactions and benefit-risk assessment of enzyme replacement therapy were at the forefront of research in this field. Treatment outcomes, early diagnosis and natural history studies of rare diseases may become hotspots for future research.

**Supplementary Information:**

The online version contains supplementary material available at 10.1186/s13023-023-02915-y.

## Introduction

In 1974, the French first created the concept of pharmacovigilance (PV) [1] which meant surveillance, guarding, and readiness to deal with possible hazards from drugs. In 2002, The World Health Organization (WHO) expanded the definition of pharmacovigilance in its book *The Importance of Pharmacovigilance* to include “the science and activities relating to the detection, assessment, understanding, and prevention of adverse reactions or any other possible drug-related problems” [2]. Although the pharmacovigilance regulatory framework did not distinguish between conventional and orphan drugs, the approved of orphan drugs in recent years, and the post-marketing safety evaluation of orphan drugs present unique challenges to regulators [3]. Rare diseases (RDs) are a group of diseases with very low incidence and drugs used to treat rare diseases are collectively known as orphan drugs (ODs). The Orphan Drug Act of 1983 initially defined an orphan drug as a treatment whose development costs exceeded potential profits [4] and later expanded to include drugs for any rare disease. In addition to orphan drugs, the therapeutic landscape for rare diseases in clinical practice encompasses a range of interventions, such as off-label medications [5], drug repurposing [6], radiation therapy [7], chemotherapy [8], dietary modifications, and medical equipment, among others.

According to the European Medicines Agency (EMA), there are 6,000–8,000 known rare diseases worldwide, about 80% of which are hereditary [9]. As research into rare diseases intensifies, certain rare diseases will no longer be “rare” and new, unknown rare diseases will continue to be discovered. Despite the small number of patients with each rare disease, there are more than 350 million patients worldwide [10], and less than 10% of rare disease patients receive targeted treatment [11]. Although some rare diseases may be subject to off-label use, substantial advancements have been achieved in the recent past concerning the development of orphan drugs. For example, The European Medicines Agency has granted approval for 20 advanced therapy medicinal products (ATMPs) [12], and a plethora of ATMPs are presently in various stages of clinical trials. It is worth noting that there is still no globally accepted uniform definition of rare diseases and the definition of rare diseases varies slightly by country/region. For example, rare diseases in The United States are defined as diseases that affect less than 200,000 people or account for less than 0.75‰ of the total population. The EU defines rare diseases as those with fewer than 180,000 people or less than 5‰ of the total population [13]. WHO defines rare diseases as those with an incidence of 0.65‰ – 1‰ [14]. A disease’s classification as ‘rare’ can be geographically contingent. For instance, Tay-Sachs disease might be categorized as rare within the general populace, yet it has been found to be carried at a frequency of 1/25 amongst German Jews [15]. Factors including ethnicity and environmental considerations may thus play significant roles in the manifestation of rare diseases.

In the case of rare diseases, traditional randomized controlled trials (RCTs) are usually not feasible with control data that may be derived from historical controls [16]. At the time of approval, the safety of orphan drugs was still unknown [17]. To ensure the safety and efficacy of orphan drugs and the safe use of drugs by the public, pharmacovigilance studies on drugs for rare diseases are crucial. In recent years, an increasing number of scholars have focused on the field of pharmacovigilance for rare diseases and have published relevant studies. Using CiteSpace for bibliometric analysis of the field can not only help scholars to quickly understand the research hotspots, evolution and frontier and other information, but also lay a solid foundation for the further development of the field.

In 2018, Sardella et al. [18] conducted a review of five aspects of clinical development safety, spontaneous adverse drug reaction reporting, postapproval safety studies, formal clinical trials, epidemiological studies in sequence, following the concept of pharmacovigilance throughout the life cycle of a drug. However, there is no systematic discussion of the research progress, current status and research hotspots in the field of pharmacovigilance for rare diseases. Therefore, this paper uses the bibliometric software CiteSpace 6.1.R6 to analyze and visualize the current status, hot spots, and potential trends in pharmacovigilance for rare diseases. Firstly, relevant information is identified such as publication trends, countries, institutions, authors, and journals to determine general information about the field. Secondly, the keywords co-occurrence and cluster analysis are used to detect research hotspots, evolutionary processes and future trends.

## Materials and methods

### Search strategy and data collection

**Fig. 1:**
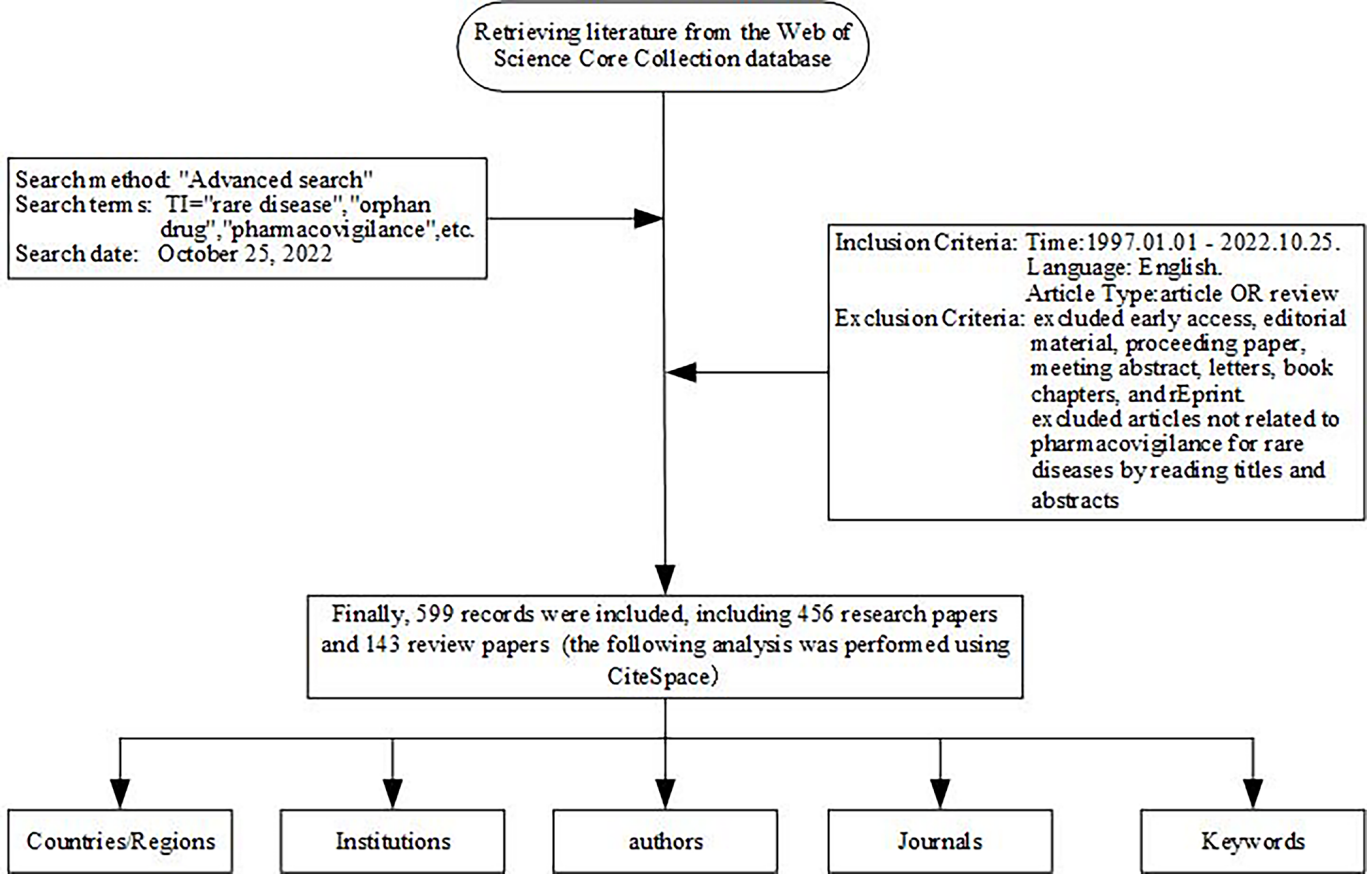
Literature search and screening

In this paper, we use the strategy of constructing a search formula to obtain the original data. Access the Web of Science Core Collection SCI-Expanded database through the official website of Guangdong Pharmaceutical University Library to search for literature. To avoid the impact of database updates, all data collection was completed on October 25, 2022. The database purchased by our unit spans the period from 1997 to the present, and the database was last updated on October 25, 2022. The data search strategy is detailed in Table 1. Initially, we retrieved 1134 publications from the year 1997 to 2022. First excluded Early Access (18), Editorial Material (12), Proceeding Paper (9), Meeting Abstract (8), Letters (5), Book Chaps. (4), and Reprint (1) to obtain 1077 publications, and then excluded articles not related to pharmacovigilance for rare diseases by reading titles and abstracts (478). In the methodology, defined as “articles not related pharmacovigilance for rare disease were excluded by reading titles and abstracts”, the inclusion criteria were articulated as follows: (1) The literature addresses adverse reactions of pharmacological treatment for rare diseases. (2) The literature focuses on safety evaluation or benefit-risk assessment of pharmacological treatment for rare diseases. (3) Study of rare diseases occurring in patients as a result of an adverse reaction to a medicine. Any literature satisfying one of these criteria was integrated into this study. Exclusion criteria: There was no mention in the literature of any studies on the therapeutic safety content of medicines for rare diseases. Finally 599 publications (Appendix 1) included including 456 research articles and 143 review articles. The detailed process of literature search and data screening is shown in Fig. 1. All the original data of 599 publications were downloaded in the format of “plain text file” → “full record with cited references”, and the downloaded text was named with “download _” at the beginning. Furthermore, given the objective of this investigation to methodically appraise and dissect the corpus of literature in the domain of pharmacovigilance for rare diseases through the employment of bibliometric techniques, this study expressly excludes consideration of grey literature, direct engagement with healthcare professionals, and related factors.

**Table 1:** Detailed search strategy in this study

Set	Result	Search query
1	31,445	(((((((TS=(“rare disease*”)) OR TS=(“rare disorder*”)) OR TS=(“orphan disease*”)) OR TS=(“infrequent disease*”)) OR TS=(“seldom disease*”)) OR TS=(“ultra-rare disease*”)) OR TS=(“orphan* drug*”)) OR TS=(“orphan* medicinal”)
2	82,125	(((TS=(“rare” NEAR/5 “disease*”)) OR TS=(“rare” NEAR/5 “disorder*”)) OR TS=(“orphan*” NEAR/5 “disease*”)) OR TS=(“orphan” NEAR/5 “drug*”)
3	82,298	#1 OR #2
4	242,617	((((((TS=(“Pharmacovigilance”)) OR TS= (“adverse drug* reaction*”)) OR TS= (“adverse reaction*”)) OR TS= (“adverse drug* effect*”)) OR TS= (“adverse event*”)) OR TS= (“adverse drug* event*”)) OR TS= (“drug* safe*”)
5	3844	((TS= (“post-marketing surveillance”)) OR TS= (“drug* surveillance program”)) OR TS= (“spontaneous report*”)
6	243,892	#4 OR #5
7	1134	#3 AND #6
8	1077	#7 AND **Language (English)** AND **Document Type** (Article OR Review)
9	599	Exclusion of 478 articles not related to pharmacovigilance for rare diseases

### Data analysis

CiteSpace is a software developed by Professor Chaomei Chen and his team for bibliometric research, which supports knowledge mining and visualization in databases [19]. Firstly, CiteSpace 6.1.R6 was used to generated countries/regions, institutions, authors, and journals co-occurrence networks in sequence to gain a better understanding of the entire research field. Secondly, keywords analysis was used to obtain keywords co-occurrence map, time-zone map and clusters map to identify research hotspots, detect the evolution of research hotspots and clarify research frontiers. Finally, the sudden increase of keywords in a short period of time was identified by keyword burst detection, which emphasized the sudden change of keywords to predict relevant research trends. When performing visual analysis, the parameters of the CiteSpace software were set as follows: (1) Time Slice: January 1997 - December 2022, 1 years per slice; (2) Selection Criteria: g-index: k = 25; (3) Pruning: select Pathfinder, Minimum Spanning Tree (MST) or none based on preliminary analysis results. Visualization mapping consists of nodes in the shape of wheel of the year and connecting lines, each node representing a unit (e.g., author, country/region, institution, etc.) and the connecting lines between the nodes representing collaboration or co-occurrence between them.

## Descriptive statistical analysis of papers in the field of pharmacovigilance for rare diseases

This part includes a discussion in three sections: (1) Annual trend of publications, with the aim of better analyzing the research intensity and publication trends in the field; (2) The analysis of scientific collaboration by countries/regions, institutions and authors aims to identify influential countries/regions, institutions and authors through three dimensions: macro, meso and micro, respectively; (3) Analysis of journals and co-cited journals, with the aim of identifying influential academic journals in the field.

### Annual trend of publications

**Fig. 2:**
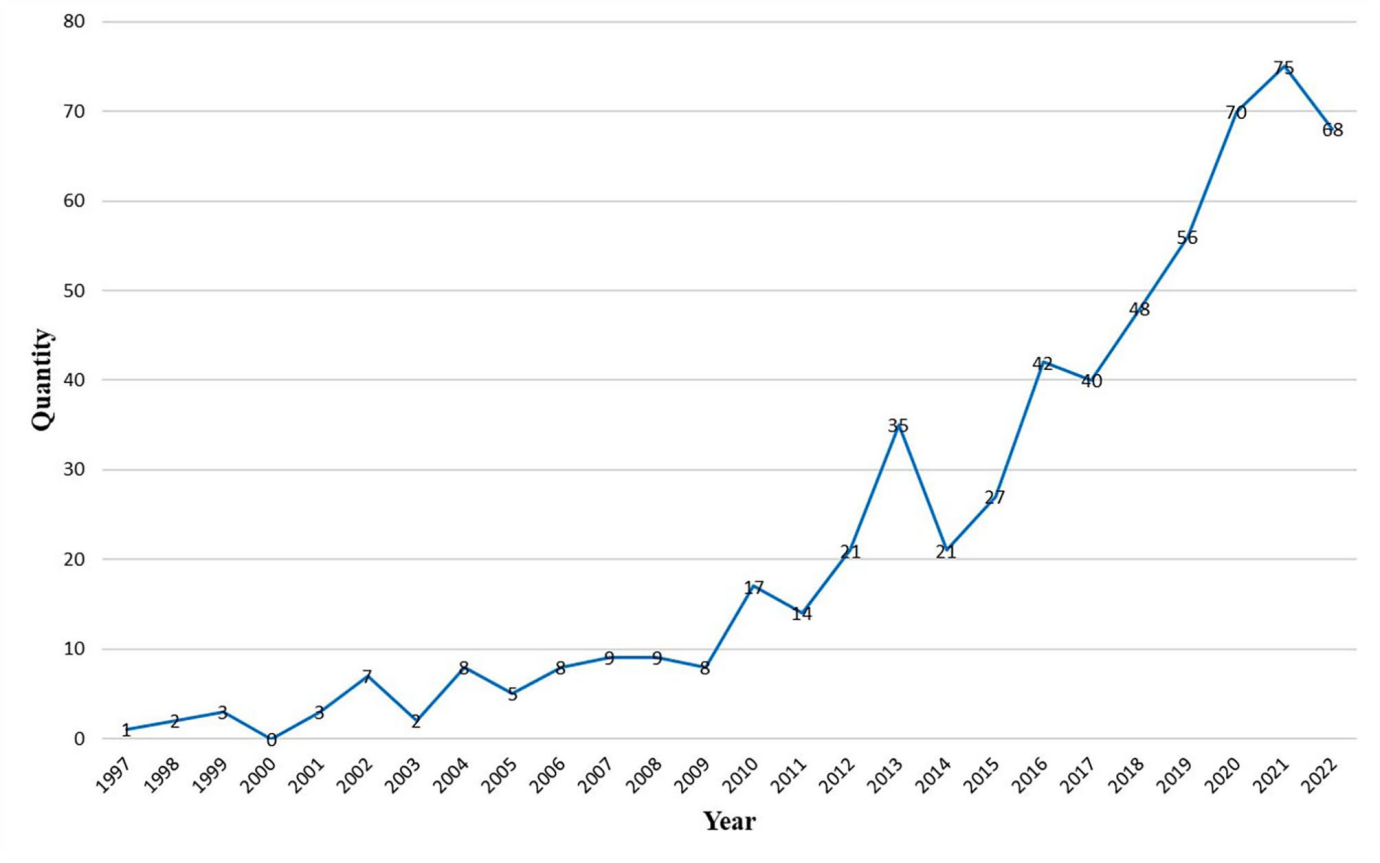
Annual output in the field of pharmacovigilance for rare diseases

From January 1997 to October 2022, 599 articles and review articles were published in the field of pharmacovigilance for rare diseases, with an average annual volume of 23 papers. Figure 2 shows that the evolution of the field of pharmacovigilance for rare diseases can be divided into three stages: start-up period (1997–2009), fluctuation growth period (2010–2015), steady development period (2016–2022).

In the start-up period, no more than 10 articles were published annually in this field. In 1997, only 1 paper was published on pharmacovigilance for rare diseases, an exploration of the specific side effects of the antiepileptic drug gabapentin [20], which was the earliest study in the field. From 1997 to 2009, the average number of articles published per year was five. 2010 was a turning point, with a significant increase in the average annual number of articles published from 2010 to 2015 (22). The annual volume of articles issued during this period shows a fluctuation increase. Production declined in 2014–2015, but not by much. From 2016 to the present, the field has entered a stable phase of development, with the average annual number of publications increasing to 57. 2021 saw the most prolific year with 75 published papers. Overall, the research in this field is not yet saturated, and the annual number of publications shows an upward trend of increasing year by year, and the number of publications will increase in the future.

### Distribution of countries/regions

**Fig. 3:**
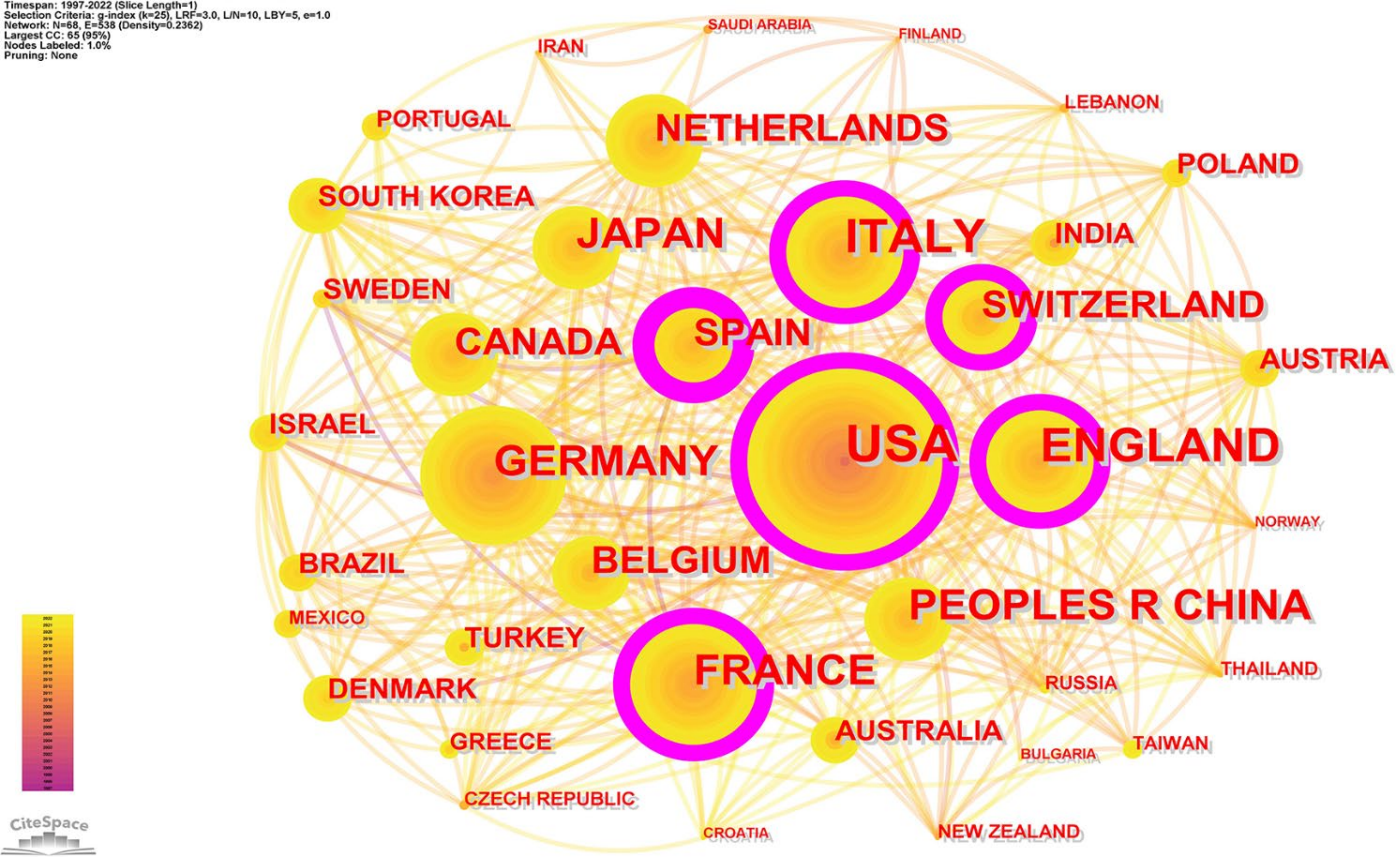
Countries/regions co-occurrence map in the field of pharmacovigilance for rare diseases

We used CiteSpace to produce a country/region co-occurrence map (Fig. 3). The map consists of 68 nodes and 538 connected lines with a network density of 0.2362, indicating that 68 countries/regions contribute to the field of pharmacovigilance for RDs. The highest number of publications was in the USA (213, 35.56%), followed by the UK (107, 17.86%), Italy (92, 15.36%), France (90, 15.03%), Germany (88, 14.69%), China (61, 10.18%) and Japan (57, 9.52%) (Table 2). The purple nodes in the co-occurrence map indicate intermediary centrality above 0.1, with Spain (0.2), the UK (0.17), France (0.15), Italy (0.13), Switzerland (0.13) and the US (0.1) playing a “bridging” role in the field.

Germany was the first country to explore pharmacovigilance for RDs (1997), followed by the United Kingdom, France and Spain (1998), the United States (1999), Italy (2001) and Australia (2002). It is worth noting that China research in this field started late (2012), however, China ranks sixth in terms of production. Considering a combination of volume of publications and intermediary centrality, the US and UK have contributed the most to the field of pharmacovigilance for rare diseases.

**Table 2:** The top 11 active countries/regions

Rank	Country/Region	Publications	Centrality	First Year
1	USA	213	0.1	1999
2	ENGLAND	107	0.17	1998
3	ITALY	92	0.13	2001
4	FRANCE	90	0.15	1998
5	GERMANY	88	0.04	1997
6	PEOPLES R CHINA	61	0.04	2012
7	JAPAN	57	0.01	2005
8	NETHERLANDS	51	0.09	2007
9	SPAIN	49	0.2	1998
10	CANADA	45	0.07	2008
11	SWITZERLAND	35	0.13	2005

### Distribution of institutions

**Fig. 4:**
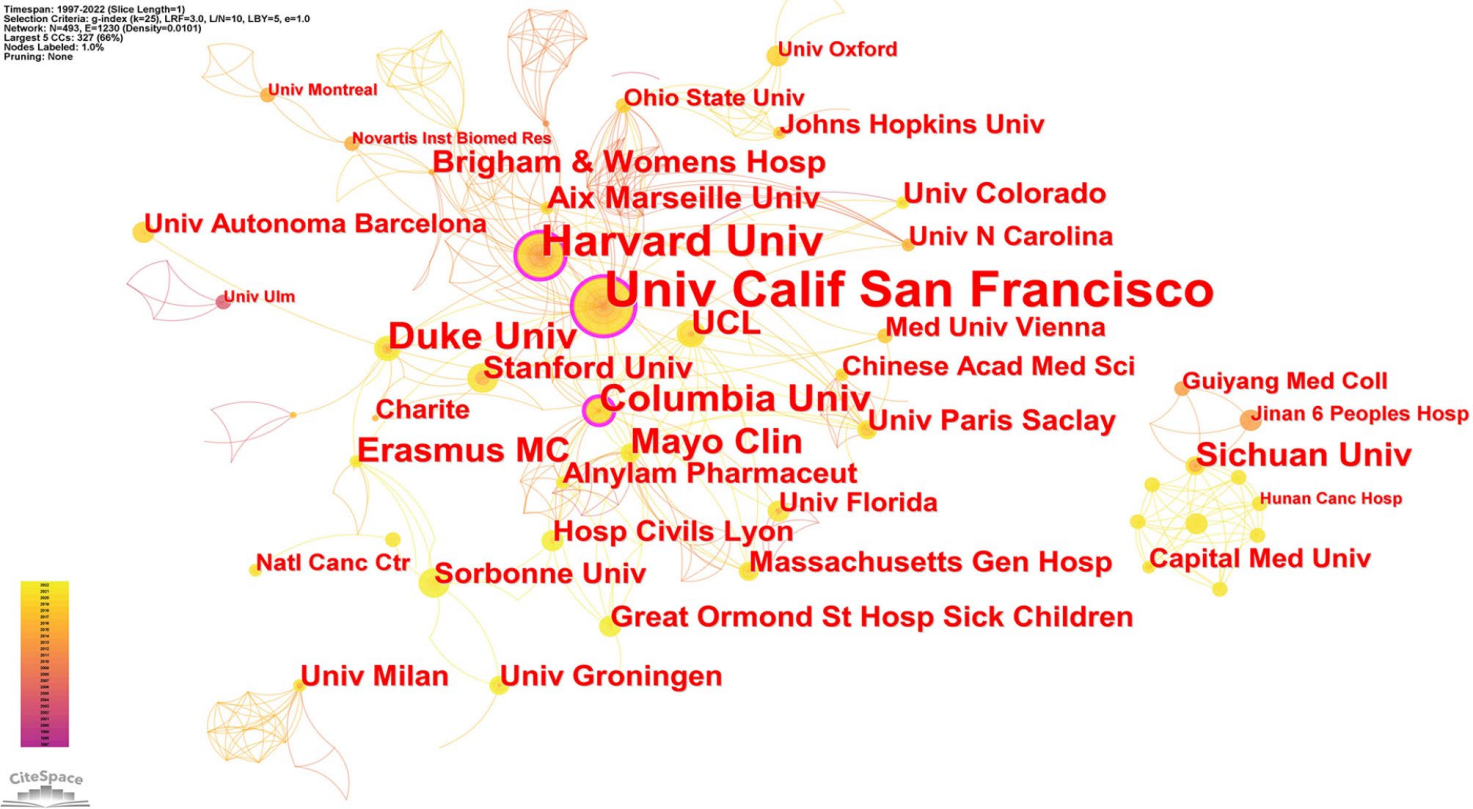
Institutions co-occurrence map in the field of pharmacovigilance for rare diseases. (k = 5)

A total of 493 institutions participated in studies of pharmacovigilance for RDs. The University of California (31) was the institution with the highest number of articles, followed by Harvard University (19), Duke University (12), Columbia University (12), and Mayo clinic (10) (Table 3). The top 11 institutions were from the US (7/11), the Netherlands (1/11), China (1/11), the UK (1/11) and France (1/11). As shown in Fig. 4, the institutional co-occurrence network includes 493 nodes and 1230 connections, and the density is 0.0101. The University of California had the highest centrality (0.2), followed by Harvard University (0.14) and Columbia University (0.11). In addition, institutions from China such as Sichuan University, Guizhou Medical University (formerly Guiyang Medical College) and Capital Medical University had contributed to the field and had formed a relatively dense network of collaborations in co-occurrence mapping. Both the University of California and Harvard University were among the top two institutions in terms of volume of publications and intermediary centrality, meaning that they had made outstanding contributions to the field of pharmacovigilance for RDs.

**Table 3:** The top 11 active institutions

Rank	Institutions	Publications	Centrality	First Year
1	Univ Calif San Francisco	31	0.2	2003
2	Harvard Univ	19	0.14	2002
3	Duke Univ	12	0.06	2010
4	Columbia Univ	12	0.11	2013
5	Mayo Clin	10	0.09	2007
6	Erasmus MC	9	0.06	2010
7	Sichuan Univ	8	0	2013
8	UCL	8	0.04	2016
9	Aix Marseille Univ	7	0.02	2014
10	Brigham & Womens Hosp	7	0.01	2010
11	Stanford Univ	7	0.03	2016

### Authors

**Fig. 5:**
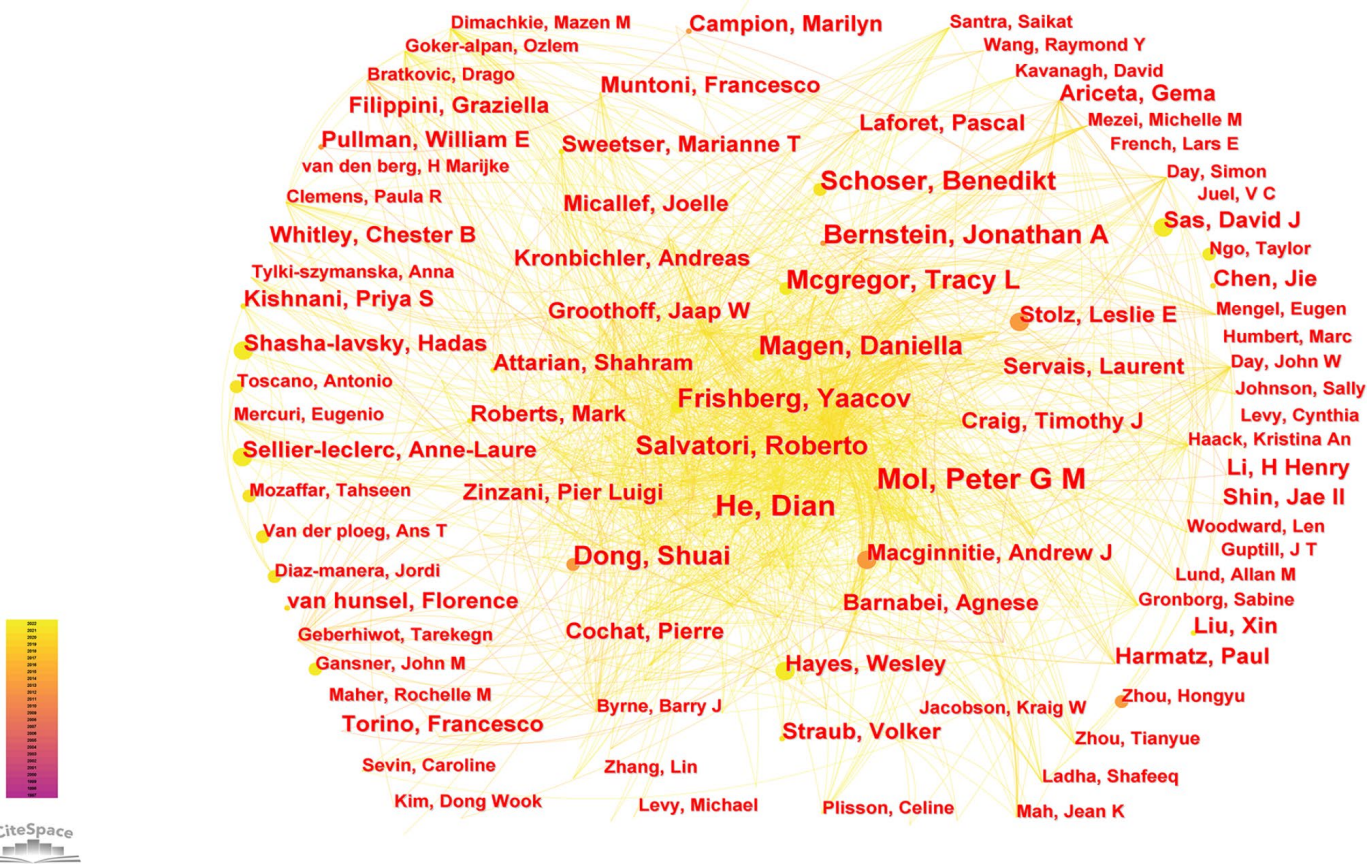
Author co-occurrence map in the field of pharmacovigilance for rare diseases

Econometricians Katz and Martin in 1997 defined “scientific collaboration” as the cooperation of scholars for a common scientific goal [21]. Scientific collaboration can take the form of publishing articles together. A co-occurrence analysis was performed by CiteSpace in the RDs pharmacovigilance domain (Fig. 5), and the co-occurrence network included 4808 nodes, 14,851 connections, and a density of 0.0013. This indicates that 4,808 scholars had contributed to the pharmacovigilance of RDs. He Dian from the Affiliated Hospital of Guizhou Medical University, China, and Peter G.M. Mol from the University of Groningen, the Netherlands, were the most published scholars (n = 5) (Table 4). He Dian and her team were dedicated to the study of multiple sclerosis treatment. They had systematically summarized and studied the safety, tolerability and efficacy of teriflunomide [22], dimethyl fumarate [23], rituximab [24] and laquinimod [25] in the treatment of multiple sclerosis. “Disease registration” [26, 27], “regulatory decision making” [28, 29] and “adverse reaction reporting” [30] have been the main directions of research by Peter G.M. Mol’s research team in recent years. Their latest study was a disease registry of heterozygous cerebral leukodystrophy using the Delphi method with a consensus initiative [27]. Nodes with high centrality tend to play an important role in the development of the scientific field, but the centrality of authors in the author co-occurrence map of the field was generally lowly (Centrality < 0.1), indicating that scholars need further collaboration in the research process of the field. It was worth noting that although the number of publications per author was lowly, a large number of scholars had paid attention to the field and published related articles, indicating that the field has good prospects for future development.

**Table 4:** The top 9 active authors

Rank	Author	Institution	Country	Publications	Year
1	Peter G.M. Mol	University of Groningen	Netherlands	5	2010
2	Dian He	Affiliated Hospital of Guiyang Medical College	China	5	2013
3	Benedikt Schoser	University of Munich	Germany	4	2021
4	Tracy L Mcgregor	Alnylam Pharmaceuticals	USA	4	2021
5	Roberto Salvatori	Johns Hopkins University	USA	4	2012
6	Daniella Magen	Ruth Children’s Hospital	Israel	4	2021
7	Shuai Dong	Jinan No.6 People’s Hospital	China	4	2013
8	Yaacov Frishberg	Shaare Zedek Medical Center	Israel	4	2021
9	Jonathan A Bernstein	University of Cincinnati	USA	4	2011

### Journals and co-cited journals

A total of 388 academic journals published 599 articles related to pharmacovigilance for RDs, with the Cochrane Database of Systematic Reviews being the most published journal (31 articles), followed by Orphanet Journal of Rare Diseases (22 articles) and Haemophilia (14 articles), meaning they had an important role in the field of pharmacovigilance for rare diseases. Table 5 listed the top 10 journals in terms of number of publications, of which 4 (Cochrane Database of Systematic Reviews, Drug Safety, The Lancet Neurology, and Lancet) journals were in the Q1 partition and 3 had IF above 10.0. There were also a number of papers published in the world’s leading journals such as The Lancet Neurology and Lancet in the field, and these high-quality papers may provide the theoretical basis for future research.

**Fig. 6:**
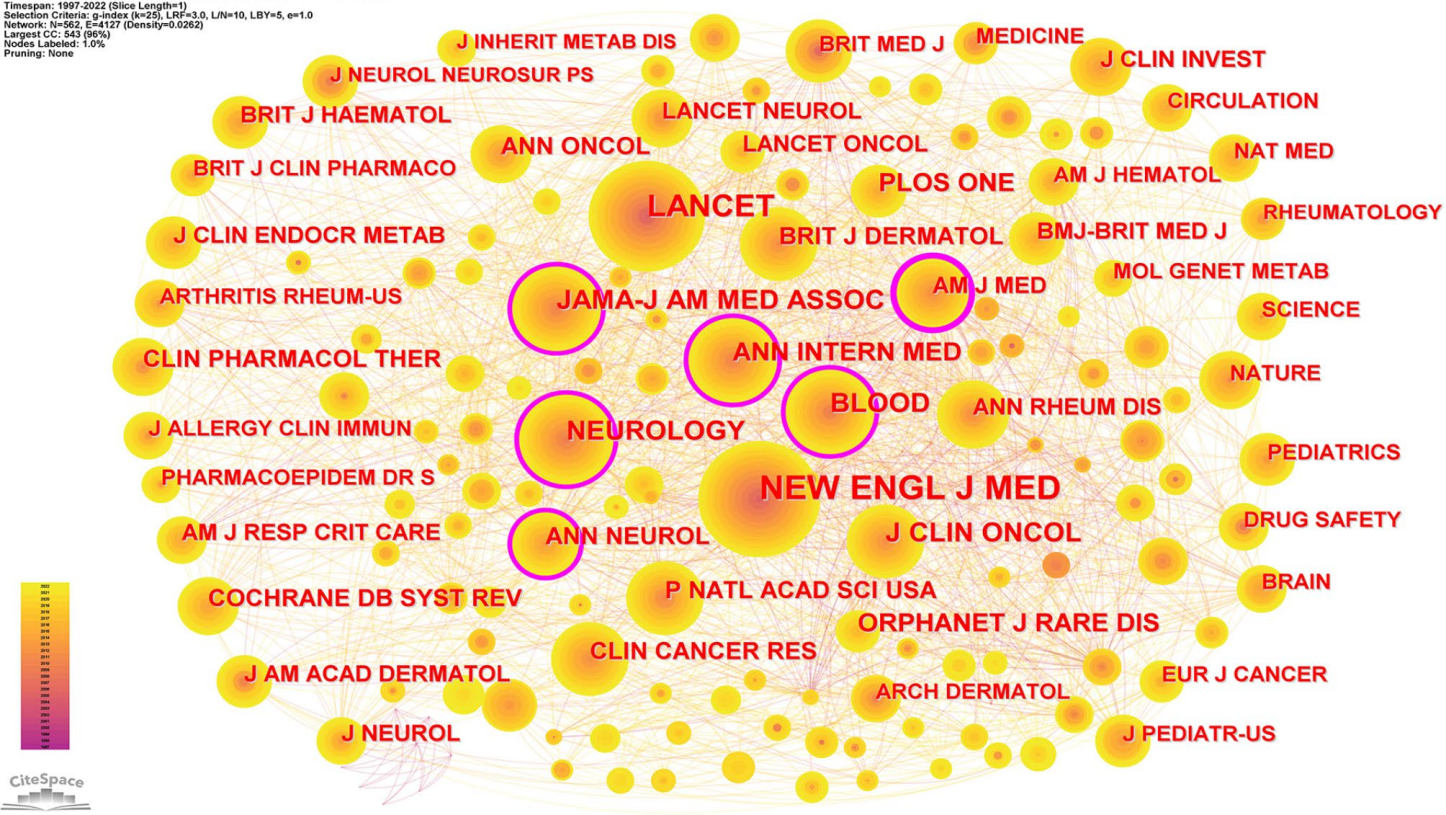
Co-cited journals

CiteSpace was used to produce a network map of co-cited journals (Fig. 6) and identified a total of 562 co-cited journals. The New England Journal of Medicine was the most cited journal in total (325), followed by The Lancet (298), Neurology (107) and Blood (103). Table 5 listed the top 10 cited journals in terms of frequency of citations. The average IF score of the top 10 co-cited journals was 30.120, and the field was expected to publish more high-quality articles in the future based on the support of high-quality references in these top journals.

**Table 5:** The top 10 popular journals and cited journals

Rank	Journal	Count	IF	Co-cite Journal	Count	IF
1	Cochrane Database of Systematic Reviews	31	12.008	NEW ENGL J MED	325	176.079
2	Orphanet Journal of Rare Diseases	22	4.303	LANCET	200	202.731
3	Haemophilia	14	4.263	NEUROLOGY	107	11.800
4	Drug Safety	9	5.228	BLOOD	103	25.467
5	The Lancet Neurology	9	59.935	J CLIN ONCOL	98	50.717
6	Expert Opinion on Drug Safety	7	4.011	JAMA-J AM MED ASSOC	94	157.335
7	Pharmacoepidemiology and Drug Safety	7	2.732	PLOS ONE	79	3.752
8	The Lancet	6	202.731	ANN INTERN MED	74	51.598
9	Medicine	6	1.817	ORPHANET J RARE DIS	71	4.303
10	Vaccine	6	4.169	ANN NEUROL	61	11.274

## Main research hot spots in the field of pharmacovigilance for rare diseases

Analysis of keywords allows the identification of popular research field, research hotspots, the evolution of hotspots, research frontiers, and future trends [31]. The following was the analysis of the co-occurrence, time-zone, cluster and burst maps of keywords respectively.

### Main research directions in the field of pharmacovigilance for RDs: keywords co-occurrence analysis

**Fig. 7:**
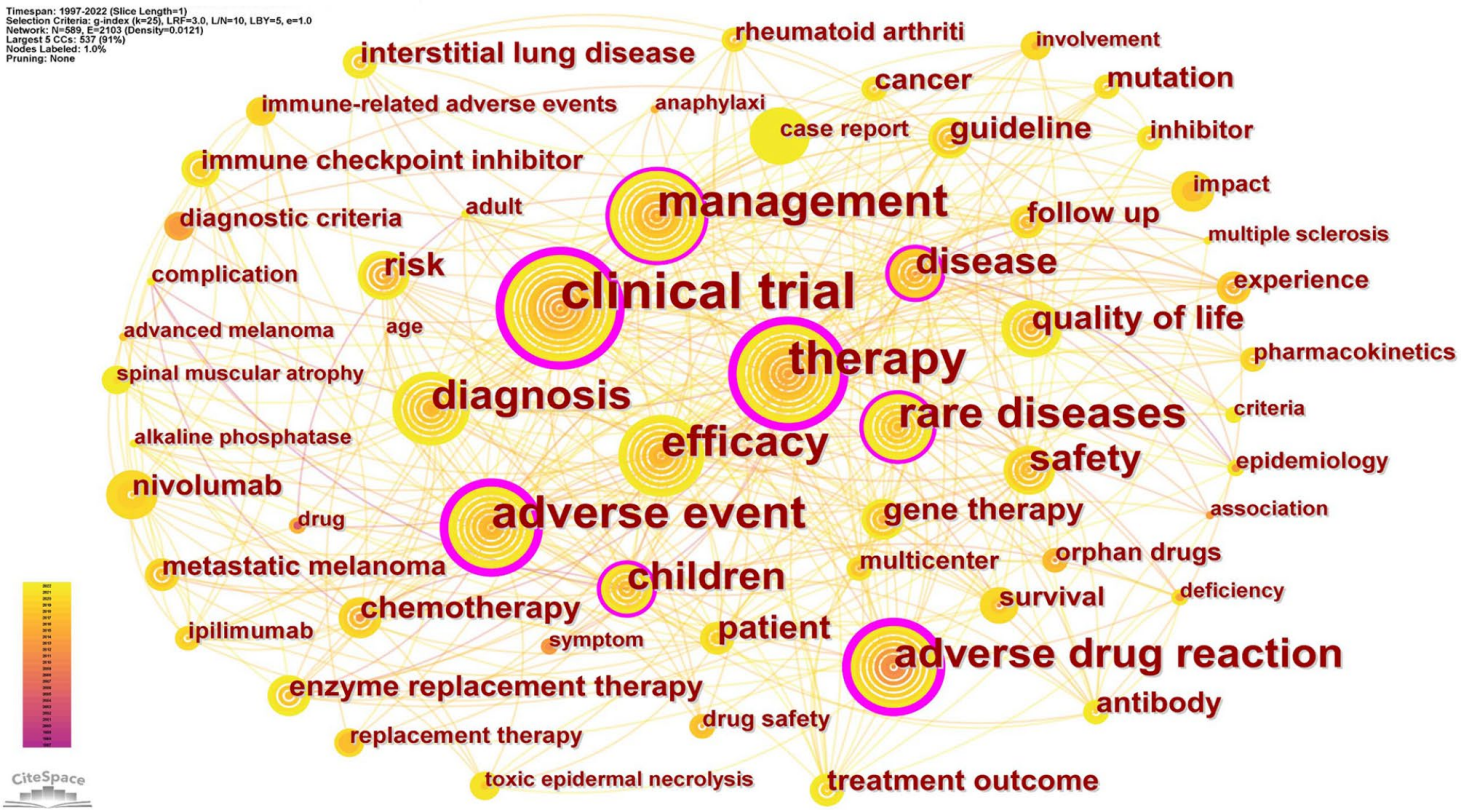
Co-occurrence knowledge map of keywords of pharmacovigilance for rare diseases

Keywords are a high level summary of an article’s research field, and the co-occurrence analysis of high-frequency keywords makes it possible to reveal a field’s relevant research hot spots [32]. A keyword co-occurrence map was constructed using CiteSpace (Fig. 7). Figure 7 shows that the eight highest frequency keywords with high centrality were: clinical trial (Frequency = 101, Centrality = 0.37); therapy (F = 62, C = 0.23); adverse event (F = 52, C = 0.32); management (F = 51, C = 0.15); rare diseases (F = 45, C = 0.11); adverse drug reaction (F = 39, C = 0.23); children (F = 30, C = 0.14) and disease (F = 27, C = 0.12). Table 6 listed the top 25 keywords with the highest frequency. Clinical trials (101) was the most frequently occurring keyword, followed by therapy (62), adverse events (52) and management (51). Based on both the criteria of frequency and betweenness centrality, this study extracted four research hotspots: clinical trials of orphan drugs, postmarketing ADR surveillance for orphan drugs, management of rare diseases and orphan drugs, diagnosis and therapy of rare diseases. What follows is a discussion of the respective four research hotspots.

**Table 6:** The top 25 high-frequency keywords

Rank	Keywords	Count	Centrality	Rank	Keywords	Count	Centrality
1	clinical trial	101	0.37	14	patient	15	0.03
2	therapy	62	0.23	15	chemotherapy	14	0.02
3	adverse event	52	0.32	16	gene therapy	13	0.06
4	management	51	0.15	17	guideline	13	0.02
5	rare diseases	45	0.11	18	enzyme replacement therapy	12	0.01
6	efficacy	43	0.09	19	interstitial lung disease	11	0.04
7	adverse drug reaction	39	0.23	20	mutation	10	0.05
8	diagnosis	39	0.09	21	antibody	10	0.02
9	children	30	0.14	22	treatment outcome	10	0.02
10	disease	27	0.12	23	cancer	10	0.01
11	safety	24	0.04	24	follow up	10	0.01
12	risk	17	0.05	25	nivolumab	10	0
13	quality of life	17	0.03				

#### Clinical trials of orphan drugs

Pharmacovigilance permeates the entire life cycle of pharmaceuticals. As delineated by the World Health Organization, the realm of pharmacovigilance encompasses every facet related to the discernment, evaluation, comprehension, and prevention of drug safety information. Consequently, data pertaining to safety and efficacy harvested during the clinical trial phase of orphan drugs constitute a crucial element in the benefit-risk assessment within pharmacovigilance for rare diseases. Presently, a plethora of orphan drugs are under the clinical trial phase, while only a minority have successfully navigated to marketing approval [33]. The probing of the efficacy and safety of orphan drugs during the clinical trial phase has emerged as a focal research point within this domain. This trend underscores the nascent nature of pharmacovigilance for rare diseases a field yet to be thoroughly explored.

Double-blind, randomized controlled clinical trials stand as the canonical standard for assessing the efficacy and safety of novel pharmacological agents. Nevertheless, the stringent adherence to classical design principles may preclude many orphan drugs from obtaining marketing authorization. Legislative measures across various nations, including the Orphan Drug Act, (EC) No.141/2000, and the Orphan Drug Management System, have facilitated the relaxation of clinical trial standards for orphan drugs. Such an adjustment is aptly illustrated by the constraints imposed by the limited prevalence of many rare diseases often only affecting a few thousand individuals rendering the conventional design of large-scale trials implausible. Pivotal trials for orphan drugs may range from a mere dozen to hundreds of participants, seldom employing single-blind, double-blind, or random-assignment methodologies, and regularly relying on surrogate endpoints [34]. Furthermore, the incidence of serious adverse events is markedly elevated in orphan drug trials, as evidenced by an American cancer study, which revealed a total of 1358 serious adverse events among 2806 trial participants receiving orphan drugs (48.4%), significantly exceeding the 1666 serious adverse events among 4621 patients in non-orphan drug trials (36.05%), with a concomitantly higher mortality rate in orphan drug trials [35].

The execution of clinical trials for therapeutic agents targeting rare diseases encounters multifaceted challenges, with the quintessential dilemma residing in acquiring ample evidence to substantiate the efficacy and safety of the drugs in question. Alongside the financial burden of conducting trials and the arduous task of enlisting adept investigators, the paramount challenge remains the procurement of an adequate patient pool suffering from rare diseases [36]. On occasion, even the amalgamation of exemplary investigators and avant-garde trial designs fails to circumvent the severe under-enrollment in randomized controlled trials for exceedingly rare conditions. To mitigate the required participant numbers in orphan drug clinical trials, an array of trial methodologies has been conceived, including Bayesian approaches, N-of-1, cross-over, sequential, and adaptive strategies, among others. Nonetheless, while each design offers tailored solutions to distinct issues, no single approach boasts universal applicability for rare diseases. Despite these manifold obstacles, the foundational ethos stipulates that every patient afflicted with a rare disease deserves access to secure and efficacious treatment, and their needs must not be overshadowed or dismissed.

Associations dedicated to the support of patients with rare diseases may serve as invaluable assets in the recruitment of participants for clinical trials. Renowned organizations such as the National Organization for Rare Disorders (NORD) within the United States and the European Organisation for Rare Diseases (EURORDIS) facilitate outreach to the afflicted patient population. Concurrently, digital platforms, such as the website hosted by the US National Institutes of Health, proffer potential trial participants access to detailed information concerning ongoing research endeavors. Furthermore, certain pharmacological agents targeting rare diseases may have achieved extensive clinical utilization over a decade or more, including but not limited to mitotane for adrenal cortical carcinoma [37], tamoxifen for Wilson’s disease, and caffeine for apnea in preterm infants [38]. In such instances, the systematic review and aggregation of extant real-world evidence may furnish a vital foundation for a robust benefit-risk assessment.

Considering that the safety profile of an orphan drug may remain relatively uncharted at the moment of regulatory approval, the acquisition of more comprehensive efficacy and safety data becomes feasible only subsequent to its wide-scale deployment in clinical practice. As a consequence, the meticulous crafting of a prospective and highly structured post-marketing surveillance program becomes indispensable in perpetuating the continuous evaluation and validation of the safety attributes of orphan drugs.

#### Postmarketing ADR surveillance for orphan drugs

Pre-market studies of orphan drug products were conducted on a very limited scale, sometimes with only a few dozen patient volunteers enrolled in clinical trials and for short study durations. Information on certain rare adverse reactions, long-term toxicity, and effects on special populations (e.g., children, pregnant women, and the elderly) was often difficult to obtain in pre-marketing studies [39]. In the pharmaceutical treatment for rare diseases, patients were often forced to accepted off-label medications because no orphan drugs were approved for the corresponding indications, and their safety was difficult to prove [40]. In recent years, certain patients with rare diseases had survived as new orphan drugs continue to be approved for marketing, but the course of these patients has never been seen, so the detection of new adverse reactions as rare diseases progress may be a result of the disease rather than an adverse reaction to orphan drugs [3], which makes monitoring for adverse reactions to orphan drugs more difficult. A correlation is discernible between the incidence of serious drug side-effects and the magnitude of a country’s population; countries with expansive populations manifest a heightened likelihood of encountering serious side-effects in contrast to nations with smaller populations, particularly within the context of post-marketing surveillance of adverse reactions to orphan drugs. Based on the rigid demand of public health for the safety of orphan drug products, post-marketing ADR surveillance of orphan drugs has become research hotspots in this field. According to the research objectives of the publications, this hotspot focused on determining the incidence of ADR and the drug-ADR association.

Representative studies that have determined the incidence of ADR include: Bürger et al. [41] research found that the overall incidence of osteonecrosis in pediatric patients with acute lymphoblastic leukemia (ALL) treated with a BFM regimen was 1.8% over 5 years, with an incidence of 8.9% in patients aged 10 years and 16.7% in patients aged 15 years. Yoneda et al. [42] identified a 1.2% incidence of interstitial lung disease (ILD) triggered by Crizotinib, with an onset time of 7-763 days, average onset time of 23 days, and recommended close monitoring for this adverse event. Representative studies that have identified drug-ADR associations include: Perros et al. [43] demonstrated that mitomycin induced pulmonary veno-occlusive disease (PVOD).

#### Management of rare diseases and orphan drugs

The management of rare diseases and orphan drugs was divided into two main aspects: on the one hand, the implementation of incentive policies to stimulate the development and production of orphan drugs in the research and development phase; On the other hand, in the field of rare diseases where medical conditions were unmet, to ensure that patients can receive safe and effective pharmacological treatment to the greatest extent possible. How to ensure that patients with rare diseases had access to medicines was the focus of such research. By far the most common approaches were government imposed incentives, accelerate review and approval, and other legal regulation, along with management of patients and drugs data for precision and rational drug use.

To improve the accessibility of orphan drugs and encourage orphan drugs research and development, many countries have legislated special management of orphan drugs and introduced a series of incentive policies. For example, in the United States, according to the Orphan Drug Act, the U.S. Food and Drug Administration (FDA) will provide written comments for non-clinical trials and clinical trials conducted for the purpose of listing; government grants and contract funds can be used to pay for clinical trials; 50% of the clinical trial costs can be claimed as tax credits; orphan drugs will be granted a seven-year marketing exclusivity period after approval for marketing; the orphan drug approval process will be simplified and the number of clinical trial cases for new drugs will be reduced, etc. [44]. The Act provided incentives for pharmaceutical companies to conduct orphan drug R&D in terms of both reducing the cost of orphan drugs development and increasing post-marketing profits. Prior to the Orphan Drug Act, there were fewer than 10 orphan drugs on the market. After decades of development, as of 2018, the FDA has accepted 4,725 orphan drugs and approved 732 orphan drugs for marketing [45]. Subsequently, the EU, Japan and other developed countries had introduced incentive policies one after another.

Research on pharmaceutical treatment for rare diseases regulatory measures also continues to emerge. For example, Kesselheim et al. [35] compared the characteristics of pivotal clinical trials for orphan drugs and non-orphan drugs review and approval and found that pivotal clinical trials for orphan drugs may be smaller in size compared to non-orphan drugs and are often non-randomized, non-blinded and surrogate endpoints to assess efficacy and safety. Similar studies include a study of post-marketing regulatory strategies for orphan drugs [46]; a study of the basis for orphan drug product approval in the EU [47]; a study of the differences in risk-benefit assessment of orphan drugs by the FDA, EMA, and the Bureau of Health, Labor, and Welfare [48]; a study of rare disease policies in China [49]; and evidence-based medicine and vaccination policies research on the safety and necessity of HPV vaccination in countries where cervical cancer is a rare disease [50].

#### Diagnosis and therapy of rare diseases

The diagnosis and therapy of rare diseases has long been a challenge that has plagued the world. A survey in China in 2020 found that each rare disease patient spends an average of 4.81 years from onset to diagnosis [51], and may experience multiple misdiagnoses and incorrect treatments in between. The field of rare diseases has been greatly improved with the increasing number of new therapies (including enzyme replacement therapy [52–54], gene therapy [55–57], immune checkpoint inhibitors, and cell therapy) being used in the clinical therapy of rare diseases. However, these new therapies are in the discovery phase and are not yet mature. Assessing the benefit-risk in the real-world of these new therapies has become a hot topic of research. Representative studies include the following. Ceulemans et al. [58] proved that low-dose fenfluramine can control the attack of Dravet syndrome for a long time and is well tolerated by 5-year follow-up. Martínez Chanzá et al. [59] demonstrated the antitumor activity and safety of cabozantinib in the treatment of non-clear cell renal cell carcinoma through a real-world study. As high-quality evidence continues to emerge, more and more orphan drugs and combination regimens will be proved with safety and efficacy.

### The evolution, frontiers and trends of the research hot spots

#### The evolution of the research hot spots: keywords time-zone map analysis

**Fig. 8:**
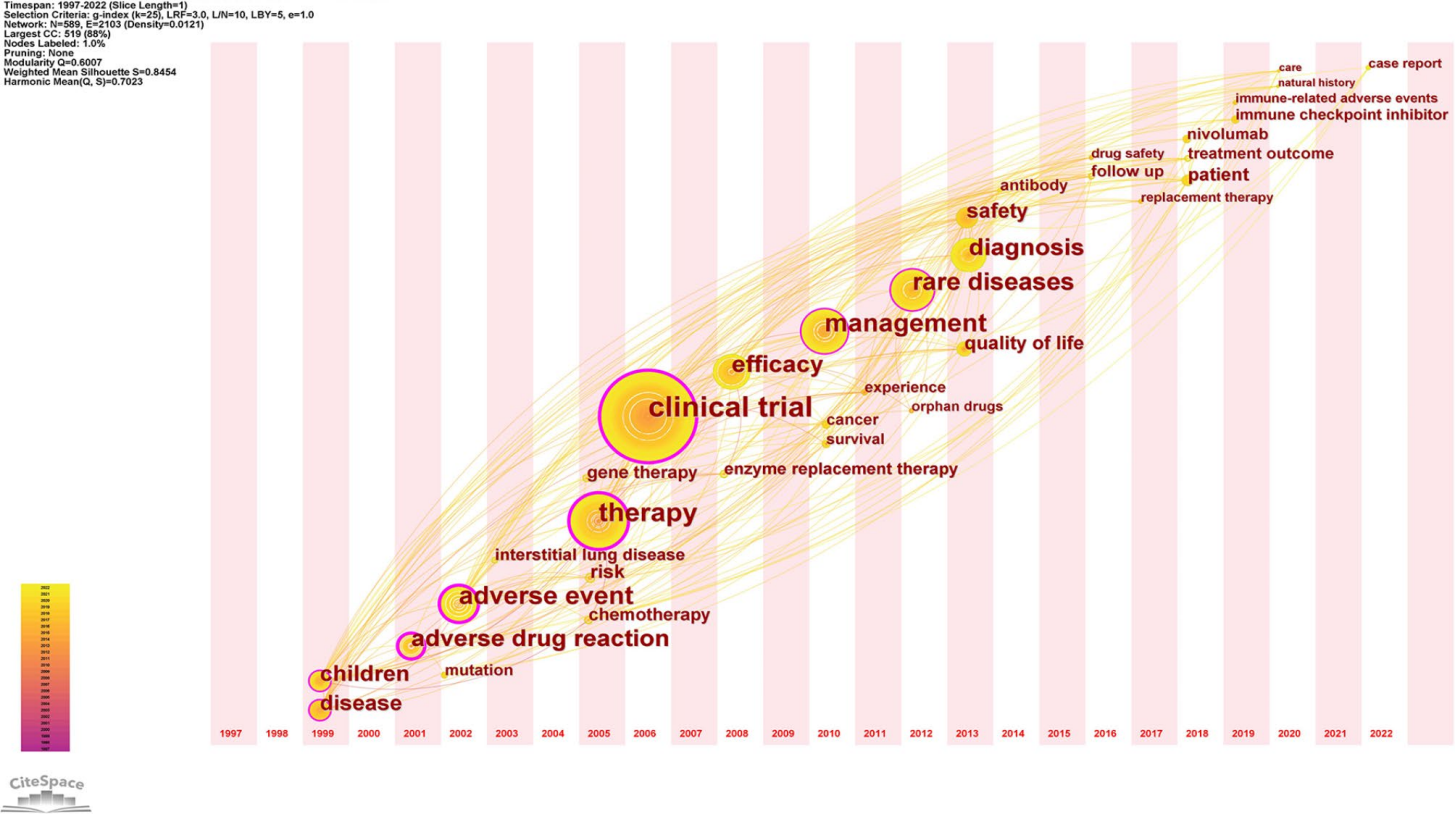
The time-zone map of keywords in the field of pharmacovigilance for rare diseases

The time-zone map produced by CiteSpace highlights the evolutionary path of research hotspots in the field of pharmacovigilance for RDs in the temporal dimension [60]. Based on the high-frequency keywords shown in Fig. 8, this study divides the evolution of research hotspots in the field of pharmacovigilance for rare diseases into four stages.

The first stage was 1997–2005. Figure 8 shows that high frequency keywords related to this stage mainly include children, disease, adverse drug reaction, adverse event and therapy. It shows that the treatment of rare diseases and the study of adverse events were the focus of research during this stage. The second stage was from 2006 to 2013, with keywords such as clinical trial, efficacy, management, diagnosis, safety and quality of life enriched the original theme. During this phase the research focus evolved to clinical trials of orphan drugs and management of orphan drug. The third stage was from 2014 to 2018. As more and more scholars focus on this field, the hot keywords for research transformed into: follow-up, drug safety, replacement therapy, patient, treatment outcome, nabumab, etc. The fourth stage was from 2019 to now. Related high-frequency keywords included immune checkpoint inhibitors, immune-related adverse events (irAEs), care, natural history, and case reports. This indicates that scholars paid more attention to the natural history and immune-related adverse events of rare diseases. The possible explanation can be that although immune checkpoint inhibitors had achieved remarkable results in the field of antitumor, the irAEs (e.g., neuromuscular toxicity, cardiotoxicity, etc.) caused by them was not increasingly negligible. The term “immune-related adverse reactions” is increasingly cited by scholars in the field of pharmacovigilance for RDs.

#### Research frontiers: keywords cluster map analysis

**Fig. 9:**
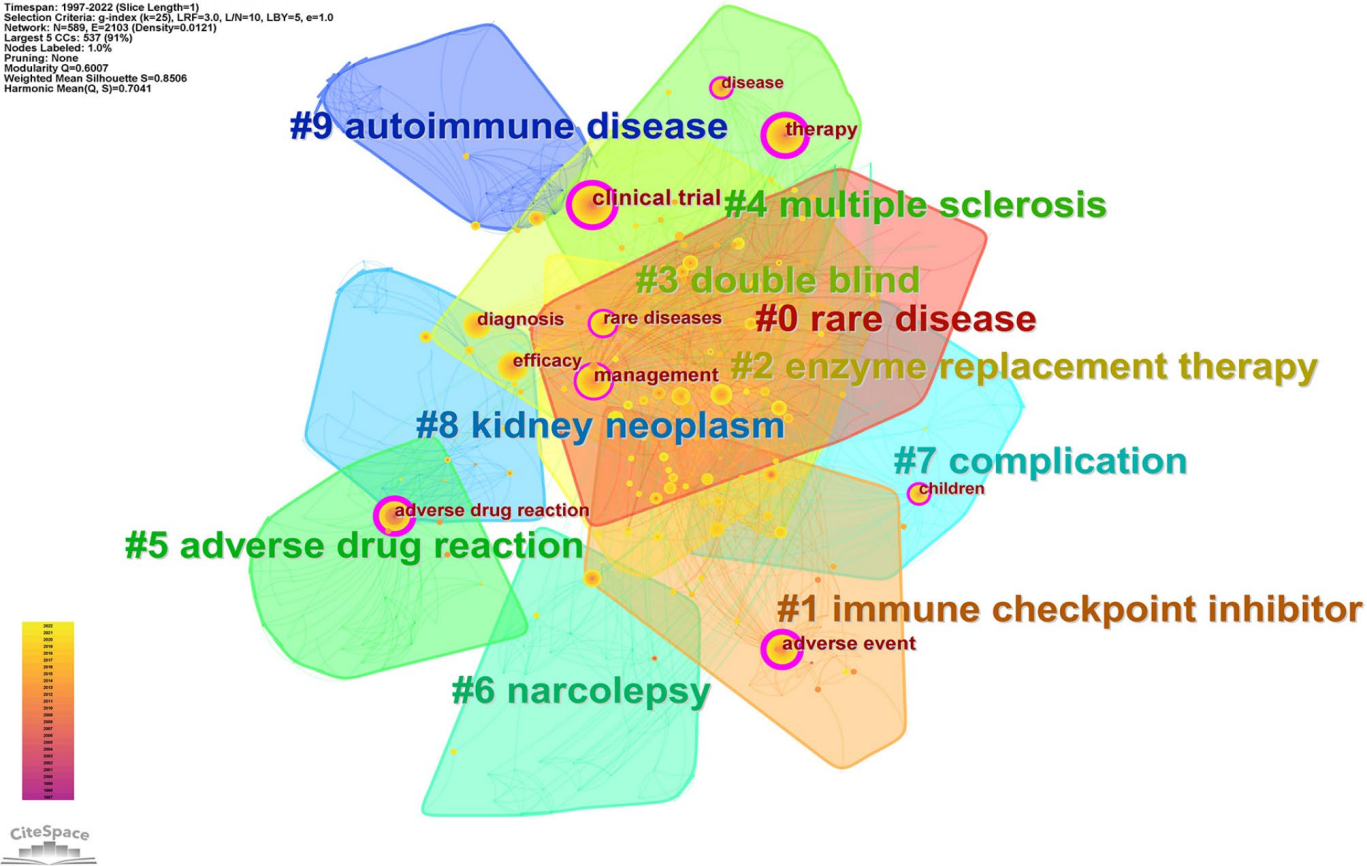
Knowledge map clusters in pharmacovigilance for rare diseases.(LSI)

The cluster analysis is the classification of keywords according to the degree of similarity of research topics and naming the clusters, which can reveal the research framework of a field [61]. In the cluster mapping Q > 0.3 indicates significant association, S > 0.5 indicates that the clustering is reasonable, and S > 0.7 indicates that the clustering is efficient and convincing. We used CiteSpace to cluster the keywords, and 589 keywords were divided into 48 clusters. Figure 9 shows the 10 largest clusters in different colors (Q = 0.6007, S = 0.8506). Figure 9 shows that rare disease, immune checkpoint inhibitor, enzyme replacement therapy, double-blind, multiple sclerosis, adverse drug reaction, narcolepsy, complication, kidney neoplasm, and autoimmune disease constituted the main knowledge structure and core research topics in the field of pharmacovigilance for RDs. Combining the research hotspots and the evolution of hotspots discussed in preceding article, we have identified two research frontiers: immune checkpoint inhibitor and enzyme replacement therapy.

Immune checkpoint inhibitors (ICIs) have shown unprecedented efficacy in treating a wide range of rare cancers, changing the outlook for the treatment of advanced rare cancer [62–64]. ICIs includes cytotoxic T lymphocyte-associated antigen-4 (CTLA-4), programmed cell death receptor 1 (PD-1, PD-L1), etc. However, when treating rare cancer, these drugs also activate immune responses in non-targeted organs, thereby inducing a wide range of immune-related adverse events (irAEs). In recent years, the study of immune-related adverse events has become a hot research direction in the field of pharmacovigilance for RDs. Numerous scholars had conducted in-depth studies on irAEs, including thyroid toxicity (destructive thyroiditis, Graves’ disease, etc.) [65], neuromuscular toxicity [62], cardiotoxicity (myocarditis, arrhythmias, etc.), celiac disease [66], autoimmune encephalitis [67], and other adverse reactions of ICIs. Most irAEs will affect the further treatment of patients to a great extent. It is very important to understand the mechanism of irAEs and to prevent and treat it as soon as possible.

Rare diseases caused by inherited metabolic disorders, such as lysosomal storage disorders, for which enzyme replacement therapy (ERT) was usually considered the standard treatment [68]. ERT is a method for the treatment of diseases based on specific enzymes produced by regular intravenous injection of recombinant DNA to replace the missing enzymes in patients [69]. As the field of pharmacovigilance for rare diseases evolves, assessing the benefit-risk of ERT has become a hot research topic. For example, Kitaoka T [70] evaluated the safety and efficacy of asfotase alfa (AA) in the treatment of hypophosphatasia (HPP), and the results of the study showed that asfotase alfa was effective in improving the symptoms of patients with HPP with a good safety profile. Additional benefit-risk studies of ERT includes vestronidase alfa for mucopolysaccharidosis VII [71]; laronidase (Aldurazyme (R)) treating mucopolysaccharidosis type I [72]; reveglucosidase alfa for late-onset pompe disease [73], etc.

#### Research trends: keywords citation bursts analysis

**Fig. 10:**
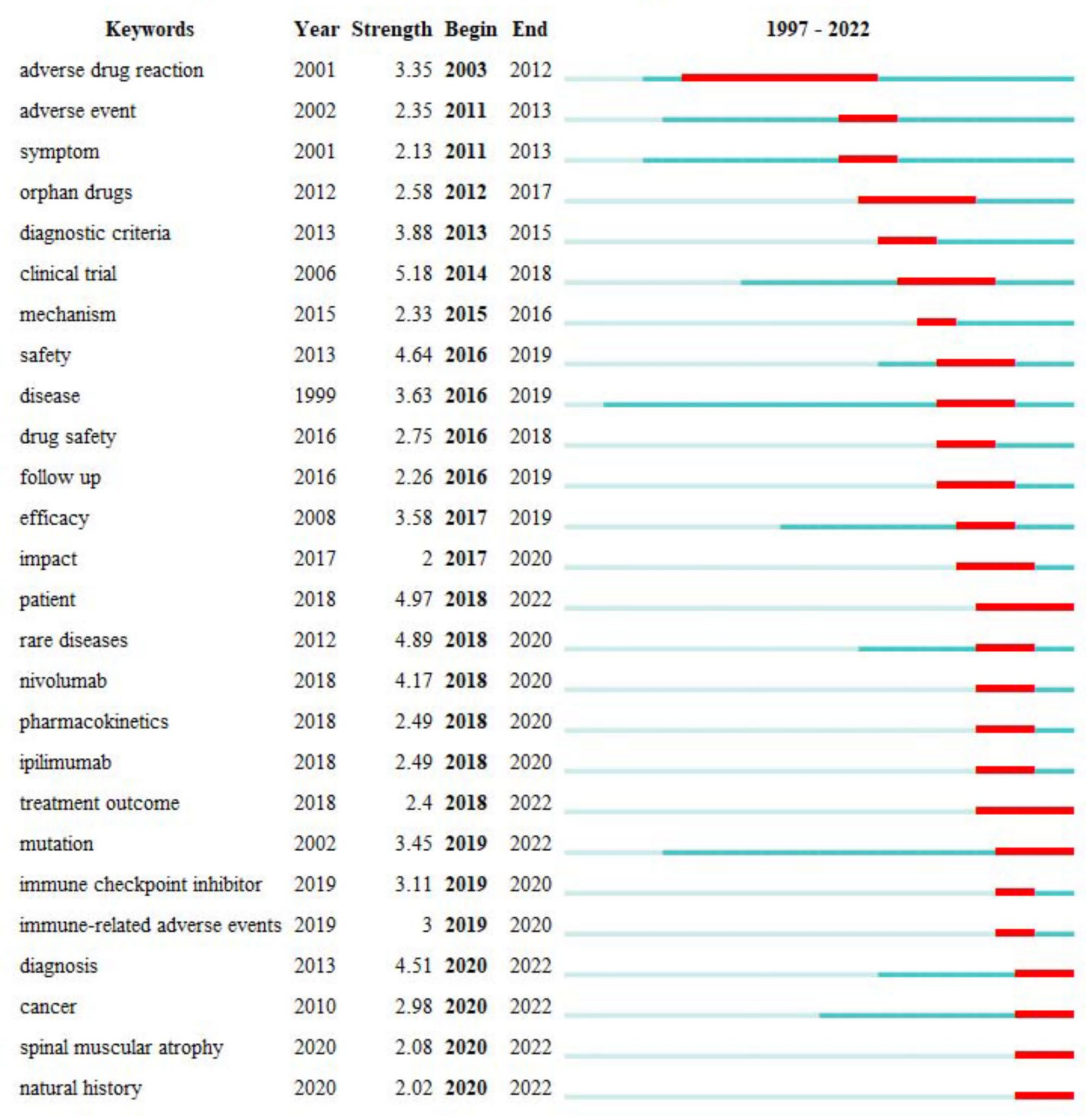
The top 26 keywords with the strongest citation bursts about pharmacovigilance for rare diseases

Keyword citation burst analysis detection can identify sudden increases in keyword citation frequency within a short period of time, revealing changes in research hotspots over time, and reflecting the evolutionary trend of research hotspots [74]. Figure 10 shows the 26 keywords with the strongest citation bursts in the field from 1997 to 2022. Clinical trial, patient, diseases, safety, diagnosis, and nivolumab were the six keywords with the strongest citation bursts, all with burst strength of more than 4.0. The longest duration of burst was “adverse drug reaction”, which lasted for 10 years. Keywords that high-intensity citation bursts for 2022 include patient, treatment outcome, mutation, diagnosis, cancer, spinal muscular atrophy, and natural history. This study considers treatment outcomes of patients with rare diseases, early diagnosis, and natural history of disease research as future research trends in the field based on high-intensity citation burst keywords in 2022 (i.e., citations spanning to 2022).

## Discussion

The domain of pharmacovigilance in the context of rare diseases has demonstrated a marked upward trajectory in the annual volume of scholarly publications spanning the years 1997 to 2022, thereby evidencing sustained academic engagement within this specialized field. A geographical co-occurrence analysis illuminates the close collaborative ties among various nations and regions. This collaboration might be attributed to the inherently small patient population for individual rare diseases and the pronounced variability exhibited across different geographical regions [75]. Such diversity necessitates cross-national and regional studies to rigorously validate the safety and efficacy of orphan medicines across disparate countries, ethnic groups, and regions. A survey of the leading institutions in terms of publication output reveals that six out of the top ten are based in the United States, signifying the country’s preeminence in the global landscape of research on pharmacovigilance for rare diseases. An examination of author co-occurrence mapping, however, uncovers a lack of an expansive author collaboration network within the field. This could be potentially attributed to the intricate nature of rare diseases’ pathogenesis, predominantly gene-related, and the considerable disparities existing between various rare diseases, coupled with a dearth of foundational research. Such complexity poses challenges for academic collaboration among researchers exploring different rare diseases. Identification of core journals with notable output serves a dual purpose: it not only facilitates researchers in accessing a comprehensive and authoritative knowledge base but also assists them in strategically selecting outlets for their submissions [76]. Within this context, the Cochrane Database of Systematic Reviews and the Orphanet Journal of Rare Diseases emerged as the most productive journals, with the New England Journal of Medicine being the most frequently cited.

Analysis of keyword co-occurrence and clustering can elucidate current research focal points, emerging frontiers, and prevailing trends in the realm of pharmacovigilance for rare diseases. As evidenced by the recurrence of high-frequency keywords, the clinical trials of orphan drugs, post-marketing adverse drug reaction (ADR) surveillance for orphan drugs, management protocols for rare diseases and orphan drugs, as well as diagnosis and therapeutic strategies for rare diseases, constitute the principal research hotspots within this field. It is imperative to underline that this area of inquiry remains relatively uncharted and calls for more profound and dedicated exploration by scholars in the future.

Through a meticulous clustering analysis of key terms, we discerned that the knowledge structure within the sphere of pharmacovigilance for rare diseases constitutes a multi-centered complex system, interconnected through various sub-domains. This intricate framework can be articulated through the following salient aspects:

### (1) Diagnosis and medication management of rare diseases

The pervasive issue of misdiagnosis acts as a significant barrier to timely and precise medical intervention for patients afflicted with rare diseases. Such misdiagnoses frequently culminate in erroneous medication prescriptions, consequent treatment delays, and adverse events. Minimizing the misdiagnosis rate of rare diseases and fortifying medication management practices are imperative to mitigating the incidence of adverse reactions.

### (2) Clinical trial studies of orphan drugs

The purview of pharmacovigilance envelops the entire lifecycle of pharmaceuticals. During clinical trial phases, sponsors are obligated to constitute a robust pharmacovigilance system designed to assiduously amass information pertinent to drug safety and ensure the welfare of human subjects.

### (3) Investigation of adverse reactions and risk factors of orphan drugs

This constitutes a vital component of pharmacovigilance and entails an exhaustive exploration of orphan drugs’ side effects and the concomitant risk factors.

### (4) Benefit-risk analysis of enzyme replacement therapy (ERT)

ERT, predicated on recombinant DNA technology, facilitates the synthesis of specific enzymes to supplement or substitute deficient enzymes in patients’ bodies for therapeutic purposes. The real-world evaluation of the benefit-risk balance of ERT pharmaceutical products has burgeoned into a cutting-edge area of focus within the pharmacovigilance domain for rare diseases.

### (5) Management and treatment of pediatric patients and cancer-related issues

A significant portion of rare diseases manifests during childhood, with a third of afflicted children having a life expectancy not exceeding five years of age [77]. Intensifying research, coupled with the advancement of early diagnostic and therapeutic strategies for pediatric patients with rare diseases, can substantially attenuate disease progression and enhance overall quality of life. Furthermore, the burgeoning utilization of immune checkpoint inhibitors (ICIs) in cancer therapy and the resultant emergence of immune-related adverse events (irAEs) have become contemporary focal points in the field of pharmacovigilance for rare diseases.

### (6) Therapeutic approaches to autoimmune diseases

Multiple sclerosis stands as a quintessential example of autoimmune rare diseases. The assessment of the efficacy and safety profiles of pharmaceutical interventions designed to treat multiple sclerosis continues to captivate the research interest of many scholars within this specialized field.

From a temporal perspective, the evolution of research hotspots in the realm of pharmacovigilance for rare diseases can be attributed to two central phenomena: governmental facilitation through incentive structures and an intensifying focus on the safety of medical applications for rare diseases. The underpinnings of these evolving research hotspots may reside in the unique attributes of rare diseases and the concomitant reliance of pharmacovigilance endeavors on regulatory frameworks. Initially encompassing broader considerations of rare disease treatments and adverse events, research focal points have undergone a substantive refinement, focusing on specific areas such as orphan drug clinical trials, benefit-risk assessment of enzyme replacement therapy, the natural history of rare diseases, and immune-related adverse events. Of particular note, the advancement of immune checkpoint inhibitors, including PD-1/PD-L1, has indubitably propelled the field of rare diseases forward, marking immune-related adverse events as an emergent research trajectory within the pharmacovigilance sphere.

Turning to the exploration of research frontiers, the real-world benefit-risk assessment of ERT has emerged as an area of concentrated academic pursuit, reflecting the consistent approval of new ERT products juxtaposed with the need for extended research into the efficacy and safety of their real-world applications. Additionally, immune-related adverse reactions stand as a burgeoning research frontier. As a revolutionary class of cancer treatment, immune checkpoint inhibitors (ICIs) have radically altered the prognosis for cancer patients. Yet, with the proliferation of ICIs in clinical contexts, the immune-related adverse events (irAEs) they engender present substantial challenges to ongoing patient care. Although irAEs are typically characterized as mild-to-moderate in severity, concurrent utilization of multiple ICIs may precipitate severe irAEs, or even fatality [78, 79]. The magnitude and severity of irAEs are inherently connected with varying modes of immune checkpoint engagement, thus enabling healthcare professionals to leverage prior research for targeted prevention. Such strategies entail meticulous observation of post-medication symptoms, proactive treatment upon the manifestation of irAEs to mitigate further detriment, and consistent patient follow-up.

Furthermore, in terms of prevailing research trends, the compilation of clinical natural history data as historical controls within clinical trials has gained traction in the development of rare disease therapies. Despite inherent challenges in employing historical controls, the invocation of placebo treatment is regarded as ethically immoral in certain rare disease contexts lacking standard treatment modalities [80]. Concomitantly, the research landscape has elucidated a pronounced emphasis on early diagnosis of rare diseases, the preliminary identification of targeted therapeutic regimens, and the ultimate efficacy of pharmaceutical interventions for rare diseases. This refocused orientation, underscored by a commitment to early intervention and precision in treatment paradigms, augments the complex and multifaceted domain of pharmacovigilance for rare diseases.

## Highlights and limitations

To our knowledge, this was the first study to revealing the progress and dynamics of research in the field of pharmacovigilance for rare diseases through a bibliometric approach. This study visualizes contributors, collaborative networks, research hotspots, and future trends through CiteSpace. Scholars can quickly understand the development status of this field by reading this article, and by getting to know scholars and institutions in this field, it is expected to increase further cooperation and exchanges.

There are still some limitations in this study. Literature was retrieved only from the WoS Core Collection Datebase and did not include literature from Chinese or other English databases, which may have influenced the results somewhat.

## Conclusion

This study uses CiteSpace to conduct a bibliometric analysis of the field of pharmacovigilance for rare diseases, which can help researchers identify research hotspots and frontiers. The four most compelling research hotspots are clinical trials of orphan drugs, postmarketing ADR surveillance for orphan drugs, management of rare diseases and orphan drugs, diagnosis and therapy of rare diseases. It is worth noting that the immune-related adverse events and benefit-risk assessment of enzyme replacement therapy is the frontier of research in this field. The treatment outcomes of patients with rare diseases, early diagnosis, and natural history of rare diseases are the future research trends in this field.

### Electronic supplementary material

Below is the link to the electronic supplementary material.

**Supplementary Material 1: S1 Appendix**. A List of 599 Articles Included in this Study

**Supplementary Material 2: S2 Appendix**. A list of abbreviations

## Data Availability

All data for this study are available from the Web of Science Core Collection database. (Use the following URL: https://www.webofscience.com/wos/woscc/basic-search.)
