# Supplementary material for: Pharmacovigilance for rare diseases: a bibliometrics and knowledge-map analysis based on web of science
Source: Orphanet J Rare Dis. 2023 Sep 26;18:303. doi: 10.1186/s13023-023-02915-y (PMC10523788; doi:10.1186/s13023-023-02915-y)
Supplement: Supplementary file 2 — Supplementary Material 2: S2 Appendix. A list of abbreviations [file 13023_2023_2915_MOESM2_ESM.docx]

**Abbreviations Used**

| **Abbreviations** | **Full Names** |
| --- | --- |
| **AA** | **asfotase alfa** |
| **ADR** | **adverse drug reaction** |
| **ALL** | **acute lymphoblastic leukemia** |
| **ATMPs** | **Advanced Therapy Medicinal Products** |
| **BFM** | **Berlin-Frankfurt-Munster** |
| **CTLA-4** | **cytotoxic T lymphocyte-associated antigen-4** |
| **EMA** | **European Medicines Agency** |
| **ERT** | **enzyme replacement therapy** |
| **EURORDIS** | **European Organization for Rare Diseases** |
| **FDA** | **Food and Drug Administration** |
| **HPP** | **hypophosphatasia** |
| **HPV** | **human papillomavirus** |
| **ICIs** | **immune checkpoint inhibitors** |
| **ILD** | **interstitial lung disease** |
| **irAEs** | **immune-related adverse events** |
| **MST** | **Minimum Spanning Tree** |
| **NORD** | **National Organization for Rare Disorders** |
| **ODs** | **orphan drugs** |
| **PD-1** | **programmed cell death-1** |
| **PD-L1** | **programmed cell death-Ligand 1** |
| **PPIs** | **proton pump inhibitors** |
| **PV** | **pharmacovigilance** |
| **PVOD** | **pulmonary veno-occlusive disease** |
| **R&D** | **research and development** |
| **RCTs** | **randomized controlled trials** |
| **RDs** | **rare diseases** |
| **WHO** | **World Health Organization** |
| **WoS** | **Web of Science** |
| **WoSCC** | **Web of Science Core Collection** |
